# Duplex DNA-Invading γ-Modified Peptide Nucleic Acids Enable Rapid Identification of Bloodstream Infections in Whole Blood

**DOI:** 10.1128/mBio.00345-16

**Published:** 2016-04-19

**Authors:** Jörk Nölling, Srinivas Rapireddy, Joel I. Amburg, Elizabeth M. Crawford, Ranjit A. Prakash, Arthur R. Rabson, Yi-Wei Tang, Alon Singer

**Affiliations:** a HelixBind, Inc., Marlborough, Massachusetts, USA; b Department of Pathology and Laboratory Medicine, Tufts University School of Medicine, Clinical Immunology and Microbiology Laboratories, Tufts Medical Center, Boston, Massachusetts, USA; c Department of Laboratory Medicine, Memorial Sloan-Kettering Cancer Center, Weill Medical College of Cornell University, New York, New York, USA; d Department of Pathology and Laboratory Medicine, Weill Medical College of Cornell University, New York, New York, USA

## Abstract

Bloodstream infections are a leading cause of morbidity and mortality. Early and targeted antimicrobial intervention is lifesaving, yet current diagnostic approaches fail to provide actionable information within a clinically viable time frame due to their reliance on blood culturing. Here, we present a novel pathogen identification (PID) platform that features the use of duplex DNA-invading γ-modified peptide nucleic acids (γPNAs) for the rapid identification of bacterial and fungal pathogens directly from blood, without culturing. The PID platform provides species-level information in under 2.5 hours while reaching single-CFU-per-milliliter sensitivity across the entire 21-pathogen panel. The clinical utility of the PID platform was demonstrated through assessment of 61 clinical specimens, which showed >95% sensitivity and >90% overall correlation to blood culture findings. This rapid γPNA-based platform promises to improve patient care by enabling the administration of a targeted first-line antimicrobial intervention.

## IMPORTANCE

Bloodstream infections continue to be a major cause of death for hospitalized patients, despite significant improvements in both the availability of treatment options as well their application. Since early and targeted antimicrobial intervention is one of the prime determinants of patient outcome, the rapid identification of the pathogen can be lifesaving. Unfortunately, current diagnostic approaches for identifying these infections all rely on time-consuming blood culture, which precludes immediate intervention with a targeted antimicrobial. To address this, we have developed and characterized a new and comprehensive methodology, from patient specimen to result, for the rapid identification of both bacterial and fungal pathogens without the need for culturing. We anticipate broad interest in our work, given the novelty of our technical approach combined with an immense unmet need.

## INTRODUCTION

Bloodstream infections (BSIs) leading to septicemia account for roughly half of all U.S. hospital deaths (1). BSIs are initiated by a diverse group of bacteria and fungi, many with varied antimicrobial susceptibilities (2, 3). Clinical outcomes are primarily determined by the time required to initiate appropriate antimicrobial intervention, as prompt intervention with the appropriate antimicrobial has been shown to decrease the severity of the disease and reduce complications (4–6). Currently, targeted therapy for septic patients relies on sluggish blood cultures as a prerequisite for pathogen identification, which delays the time to identification by days (7, 8). In the absence of actionable diagnostic information, patients are empirically treated with potent broad-spectrum antimicrobial therapies, leaving the majority of patients receiving inappropriate or insufficient treatment, resulting in poor outcomes and contributing to the emergence of antibiotic resistance (2, 7, 9). While tremendous advances have been made in reducing the turnaround time needed to identify the pathogen directly from a positive culture, the overall time remains high, typically >24 to 36 h (7). Given that mortality rates increase by 7 to 9% for every hour the appropriate antimicrobial intervention is delayed, there is a clear need for pathogen identification well before culturing results become available (4, 10, 11). Modern molecular approaches have the potential to revolutionize this field; however, limitations, including lack of sensitivity, inaccurate performance, narrow coverage, and insufficient diagnostic detail, have prevented these methods from making an impact (7, 12–14).

The shortcomings of current molecular approaches in simultaneously targeting a broad pathogen panel at very low loads in a complex matrix such as whole blood can be described, to a first-order approximation, as a specificity problem, as enzymatic amplification of target sequences has been widely shown to be sensitive down to a single copy when maintained at low complexity (low multiplexing). Indeed, in cases of BSIs, for which the ability to discriminate among a large and specific set of microbial species is typically required, standard nucleic acid probes targeting highly similar DNA sequences, such as rRNA encoding genes, have proven incapable of providing high-confidence calls, primarily when the low-input loads result in a PCR amplicon level barely above random background amplification (14). As we describe here, γ-modified peptide nucleic acids (γPNAs), a novel class of artificial nucleic acid probes capable of invading duplex DNA rather than hybridizing to single-stranded DNA, have the potential to overcome the specificity problem, as they display characteristics superior to those of commonly used probes.

We present here a novel diagnostic platform that features the first successful adoption of γPNAs as a diagnostic tool for the rapid, consistent, and highly sensitive detection of BSIs directly from whole blood. The platform assays for 21 of the most clinically prevalent BSI-causing pathogens and reaches the single-CFU-per-milliliter sensitivity level for all panel pathogens due to the near-zero levels of probe-to-probe cross talk. Furthermore, our successful evaluation with clinical specimens highlights the valuable support this diagnostic platform would provide in the decision-making process for initial BSI intervention.

## RESULTS

### γPNAs as novel sequence interrogation tools for highly sensitive pathogen profiling.

**FIG 1:**
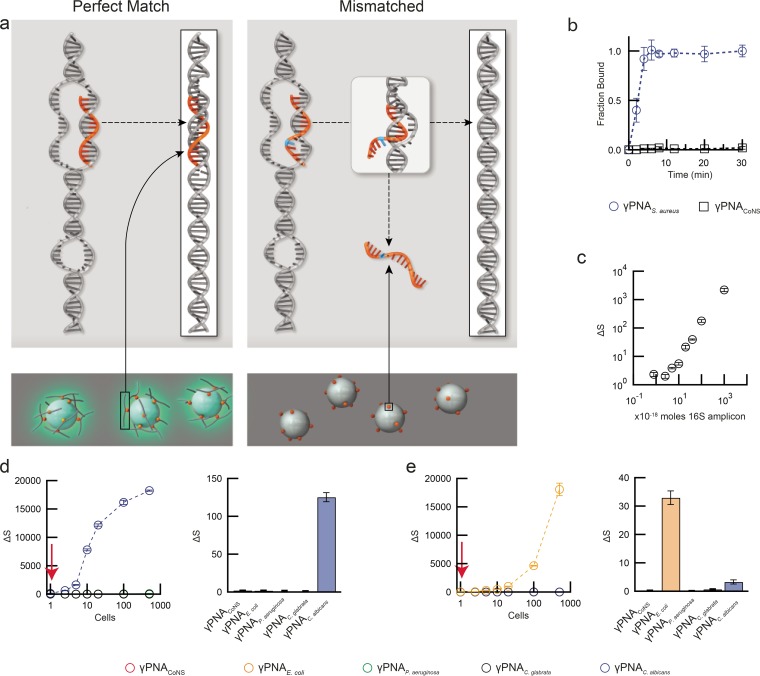
Performance characteristics of γPNAs. (a) Schematic of γPNA double-stranded DNA (dsDNA) invasion and bead capture. Locally formed dsDNA bubbles are invaded by γPNAs. Only perfectly matched γPNAs form stable hybrid structures (“triplex”), which immobilize the target amplicon onto magnetic beads, enabling optical detection (left panel) via chemiluminescence, while mismatched γPNAs are “kicked out” by the complementary DNA strand (right panel). (b) Capture kinetics of *S. aureus* 16S rRNA gene amplicon by an *S. aureus*-specific γPNA (γPNA_S.aureus_). The *S. aureus* 16S amplicon was effectively captured in <5 min by its specific γPNA, whereas a γPNA specific to *S. epidermidis* (γPNA_CoNS_) did not yield an appreciable optical signal. (c) Detection of a serially diluted *S. aureus* 16S amplicon by γPNA_S. aureus_ demonstrated detection limits of <1 amol. (d and e) Detection of serially diluted *C. albicans* (d) and *E. coli* (e) gDNA (1 to 500 cell equivalents) with both target-specific γPNAs and off-target γPNAs after 32 cycles of PCR amplification. Thirty-two amplification cycles were experimentally determined as the preferred number, as it enabled an observed detection limit of a single cell while enabling detection of significantly higher loads (left panel). The output signal at a single cell input (red arrow in left panels) underscores selectivity of γPNA target binding with high target/off-target ratios (right panels). All experiments were completed in triplicate; data are expressed as means ± SD.

We have developed a bead array-based assay that employs γPNAs, a newly developed class of duplex DNA-invading artificial nucleic acid oligomers that have significant advantages in kinetics, sensitivity, and specificity over DNA, RNA, and standard peptide nucleic acids; these capabilities are derived from the γPNA structure (15–20). First, and as in all PNAs, the negatively charged sugar-phosphodiester backbone found in DNA/RNA is replaced by a neutral *N*-(2-aminoethyl) glycine backbone, thus eliminating the electrostatic (enthalpic) penalty upon the creation of the duplex structure from two single-stranded nucleic acids via Watson-Crick base-pairing. Second, and unlike other natural and synthetic nucleic acids, γPNAs contain a stereogenic center at the γ-position of each monomer, forcing the single-stranded oligomer to preorganize into a right-handed helix. This conformation reduces the entropic penalty paid by the single-stranded oligomer during duplex formation to a complementary nucleic acid. Through the combined enthalpic and entropic advantages, γPNAs are uniquely positioned to invade duplex DNA, and unlike other artificial nucleic acids, there are no sequence restrictions. Because the invasion is local and the DNA strand complementary to the γPNA/DNA hybrid remains only a few nanometers away, competition is fierce, and if but a single mismatch is present in this hybrid, the γPNA is kicked out of the duplex DNA, as the hybrid complex is energetically unfavorable. A perfect matching γPNA, in contrast, forms a stable γPNA-DNA triplex (Fig. 1a). A consequence of this energetic competition is a high sequence selectivity, which we have exploited for diagnostic purposes in targeting a broad, but specific, set of pathogens.

γPNA oligonucleotides, 15 to 17 bases in length and specific to a bacterial or fungal DNA target, were designed through comparative analyses of either the 16S (bacterial) or 18S (fungal) sequences via BLAST (Basic Local Alignment Search Tool). Multiple sequences from each microorganism were aligned to identify both intragenomic and intraspecies conserved regions (see Table S1 in the supplemental material for γPNA sequences). Both bacterial and fungal 5′-hapten-modified PCR primers were designed to amplify full-length 16S and 18S (~1.5 kbp), as increased amplicon size has been shown (21, 22) to be efficient in reducing the effects of background contamination commonly found in broad-range 16S/18S amplification (see Fig. S1 in the supplemental material). Similar to DNA-based bead arrays, our target detection process encompasses three distinct steps: (i) γPNA-mediated immobilization of the amplified target DNA to the beads, (ii) a wash step to remove weakly bound DNA from the beads, and (iii) chemiluminescence-based detection.

Our initial studies, using a subset of our 21-target panel, established key performance characteristics of γPNAs. We first determined the kinetics at which γPNAs invade their target by conducting bead capture assays with 1 fmol of amplified *Staphylococcus aureus* 16S as the model target, using γPNA probes specific to either *S. aureus* (γPNA_S.aureus_) or to common coagulase-negative staphylococci (CoNS), such as *Staphyloccocus epidermidis* (γPNA_CoNS_). Time course studies (Fig. 1b) revealed that the *S. aureus* 16S amplicon is quickly invaded, captured, and immobilized to the beads by γPNA_S.aureus_. The process reaches saturation in <5 min, at which point >99.5% of the DNA input has been captured, a significant improvement over DNA arrays, which typically require several hours under stringent hybridization conditions (23–26). Off-target γPNA_CoNS_, in contrast, captured virtually no *S. aureus* amplicons (1.2 ± 0.1%), even after prolonged incubation, highlighting the excellent selectivity of γPNAs. Relative to common DNA-based or even standard PNA-based arrays, this significant kinetic advantage, while meeting high stringency requirements, is paramount, considering the clinical need for fast turnaround times. Notably, these kinetic characteristics are achieved while permitting excellent detection sensitivities: with the *S. aureus* 16S amplicon/γPNA_S. aureus_ combination in a titration experiment, we found that as little as 0.8 amol of target DNA (~500,000 molecules in 100 µl) can be readily detected (Fig. 1c).

We next determined the sensitivity of γPNA-mediated capture/detection linked with PCR amplification. Given the sensitivity of γPNAs, PCR conditions were optimized for both fungal and bacterial pathogen DNA to facilitate a large dynamic range rather than signal saturation (see Materials and Methods). *Candida albicans* and *Escherichia coli* genomic DNAs (gDNAs) were used to perform titration experiments over a range of 1 to 500 cells or genome equivalents, an amount hypothesized to be roughly equivalent to the pathogen load expected in clinical samples. Following amplification, a fraction of the material (~6%, simulating the amount used in a 17-plex γPNA panel described in the next section) was utilized for each γPNA-bead assay. The background-subtracted normalized signal, Δ*S* (see Materials and Methods), was calculated for both the target γPNAs and the off-target γPNAs. Results with *C. albicans* (Fig. 1d) highlighted the ability to detect a single cell with minimal probe cross talk, attaining a signal from γPNA_C.albicans_ that was >115× above the most prominent off-target signal. Similarly, single cell detection was achieved using *E. coli* gDNA as input (Fig. 1e). As expected, the signal obtained for *E. coli* at lower concentrations was less pronounced than that with *C. albicans* due to the fewer gene copies present in bacteria in relation to fungi. We note that the detection sensitivity is directly related to both high target affinity as well as the selectivity of γPNAs; off-targets are effectively removed during wash steps, thereby significantly lowering the background commonly seen in DNA-based assays. Indeed, it is the combined performance characteristics of γPNAs that enable the development of a highly effective diagnostic method.

### Species-specific detection of a comprehensive pathogen panel.

**FIG 2:**
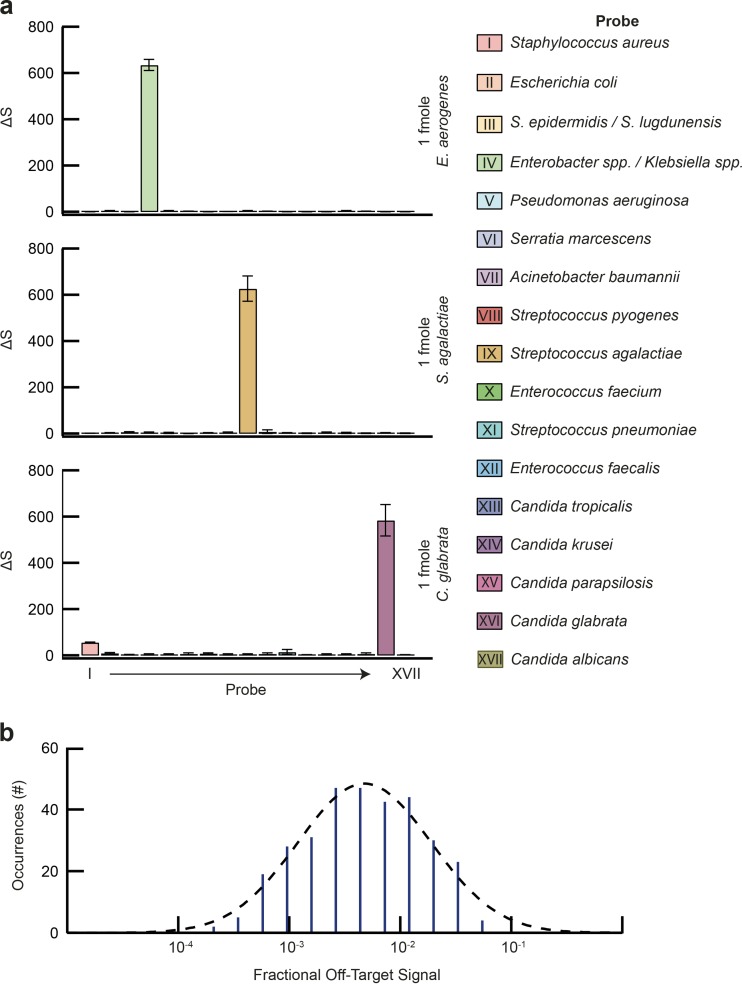
Panel γPNA probe specificity validation. The specificities of the 17 panel γPNA probes, labeled I to XVII, were evaluated against 16S/18S amplicons (1 fmol) generated from all 21 target organisms. (a) Representative results for *E. aerogenes*, *S. agalactiae*, and *C. glabrata*. Note the high signals for targeted organisms against low background signals derived from nontarget organisms. (b) Distribution of off-target signals as a fraction of target signal generated by all 17 γPNAs and all 21 panel organisms. Quantification showed a typical off-target signal of about 0.43%, indicating a typical signal/off-target ratio in excess of 200×. All studies were completed in triplicate; data are presented as means ± SD.

Targeted antimicrobial therapy requires species-level identification of the infectious agent, as the response to antimicrobials can vary significantly between highly related pathogens (2). Our diagnostic panel targets 16 bacterial and five *Candida* species, representing >91% of pathogens causing BSIs (2). To maximize coverage and provide actionable diagnostic information, a set of 15 species-specific and two group-specific γPNA probes were developed (see Table S1 in the supplemental material). Given the degree of γPNA multiplexing, probe cross talk is a concern, as this would limit sensitivity and confound results. We therefore tested and optimized the γPNA oligonucleotide sequences, as well as binding and wash conditions, for all panel pathogens. In order to assess target and background levels, rRNA encoding genes from each of the panel pathogens was amplified, and 1 fmol was tested against each of the 17 γPNAs. One femtomole was chosen as the input, given the need for a large dynamic range to determine off-target binding levels (roughly 3 logs, based on the results presented in Fig. 1). Figure 2a shows the results for three model organisms, representing a fungal pathogen (*Candida glabrata*), a Gram-negative bacterium (*Enterobacter aerogenes*), and a Gram-positive bacterium (*Streptococcus agalactiae*). Assessment of off-target binding for the entire γPNA probe set revealed a typical level of ~0.43% relative to the peak target binding signal (Fig. 2b), corresponding to a typical signal/off-target ratio of >200:1 and a maximal off-target signal of 7.1%, indicating a consistent signal/off-target ratio of >14:1 at this input level. By comparison, DNA probe-based assays described in the literature are either unable to achieve species-level detail or display markedly higher levels of background due to off-target binding (14, 26, 27).

### Direct pathogen identification from unprocessed whole blood.

We next investigated the ability to identify and discriminate pathogens directly from blood. Human whole blood is a challenging sample matrix for any diagnostic assay, particularly when processing larger volumes, as is required for sensitive detection of BSIs (8). To accentuate the advantages of γPNA-mediated pathogen identification (PID), we developed a multistep, fully integrated sample preparation process that builds and improves on traditional approaches, requiring less than 2.5 h from sample to result. The entire sample preparation methodology was developed to boost detection sensitivity by maximizing the microbial input, while focusing on extracting/purifying only high-molecular-weight gDNA, thus enabling target amplification with reduced background levels (see Fig. S1 in the supplemental material). We began with a selective lysis process, which facilitates the processing of 1.5 ml of EDTA-treated whole blood by rapidly removing >99.9% of the human DNA (hDNA) without compromising microbial cell viability. We note that this step simultaneously eliminates potential false-positive results generated from free pathogen DNA which may be present in the blood (28). Microbial cells are then subjected to chemical lysis, ensuring that the released gDNA is of high molecular weight. After completing the microbial lysis, gDNA is purified using an anion exchange system optimized to selectively capture high-molecular-weight DNA and proceeds to undergo a two-plex PCR (creating an ~1.5-kbp 16S and/or 18S amplicon) in preparation for the γPNA-mediated detection step (see Materials and Methods). Ultimately, the use of γPNAs as capture probes is markedly advantageous, as long DNA amplicons can only be immobilized through a DNA invasion process. Use of DNA capture probes is simply inefficient, as long, single-stranded DNA naturally develops numerous secondary structures which significantly lower the efficiency and specificity of the capture/immobilization process.

We proceeded to validate the integrated PID assay by using pooled human whole-blood samples spiked with pathogens at clinically relevant loads. Based on current estimates (29–31), we identified two pathogen load regimens that encompassed the majority of clinical cases: a high-load regimen with load levels between 10 and 99 CFU/ml and a low-load regimen with load levels between 1 and 9 CFU/ml. Direct-from-blood performance testing was completed for all 21 panel organisms in triplicate, and buffer-spiked blood samples were used to determine background levels in the absence of a pathogen.

**FIG 3:**
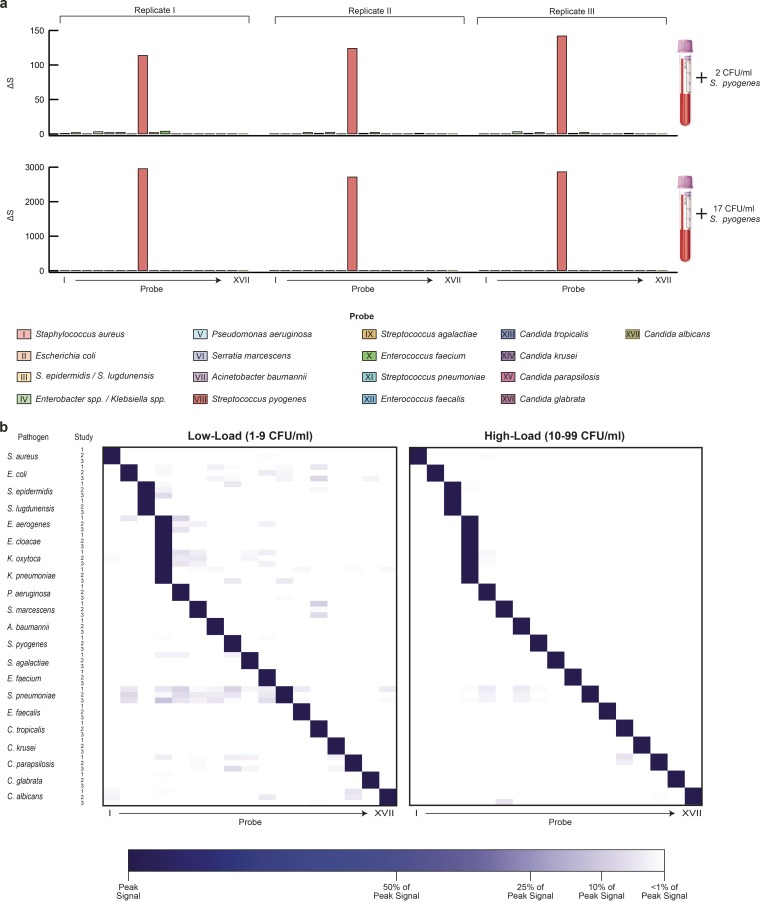
Direct blood PID validation. The performance of the integrated PID assay was tested using 1.5 ml blood spiked at both low (1 to 9 CFU/ml) and high (10 to 99 CFU/ml) pathogen loads. Processing steps included (i) removal of blood hDNA, (ii) extraction of gDNA, (iii) PCR amplification, and (iv) γPNA-based detection of amplicons. (a) Representative results for a triplicate study with *S. pyogenes* blood spiked at 2 CFU/ml (top) and 17 CFU/ml (bottom). Of the 17 γPNA probes, only probe VIII, specific for *S. pyogenes*, generated a significant signal, while signals generated from all other γPNAs were negligible. (b) Heat maps comparing results from blood spiked with either low loads (left panel) or high loads (right panel) of panel organisms. Signals were normalized to the target signal to facilitate visualization. Note that in all cases, off-target signal was negligible, even at the <10-CFU/ml level. Each pathogen/dilution combination was tested in triplicate.

Figure 3a shows the results for three replicate studies from a representative microorganism, *Streptococcus pyogenes*, at low-load (top panel) and high-load levels (bottom panel). To account for differences in rRNA gene copies, spike loads, and pathogen-specific CFU-to-cell ratios, data were normalized against the highest signal obtained for each pathogen. The normalized data for the entire panel are presented as heat maps in Fig. 3b. We note that for all targets, peak signals were above the limit of detection (see Materials and Methods). Even at the low-load level, signals for each of the 21 panel pathogens were typically 129× above their panel off-target signals. Moreover, even the highest off-target signal was still about 6× lower than the peak signal, and 98.7% of all experiments displayed at least a 10:1 signal/off-target ratio, enabling high confidence calls in all cases. These results emphasize the specificity of γPNAs and demonstrate that interference from probe cross talk is negligible.

**FIG 4:**
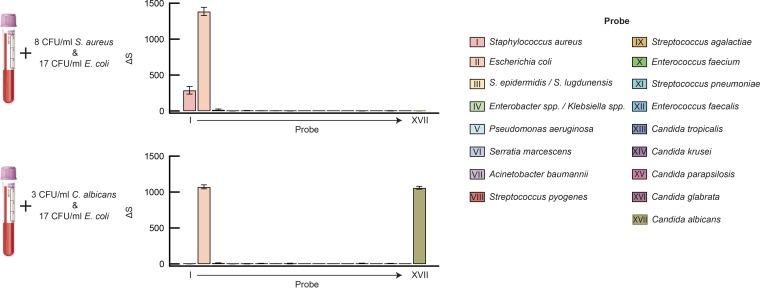
Polymicrobial detection. Blood was spiked with multiple organisms at the indicated levels. Results for *E. coli* and *S. aureus* (upper panel) and *E. coli* and *C. albicans* (lower panel) show high signal for target organisms and low signal for off-target organisms across the entire panel. Experiments were completed in triplicate; data are represented as means ± SD.

We further challenged the PID assay by processing blood spiked with two pathogens simultaneously to evaluate possible interference with target detection in the presence of polymicrobial infection. Results from both bacterium-bacterium (*E. coli* and *S. aureus*) and bacterium-fungus (*E. coli* and *C. albicans*) spike experiments showed no significant difference from the corresponding single-pathogen detections (Fig. 4).

### Performance with clinical samples.

**FIG 5:**
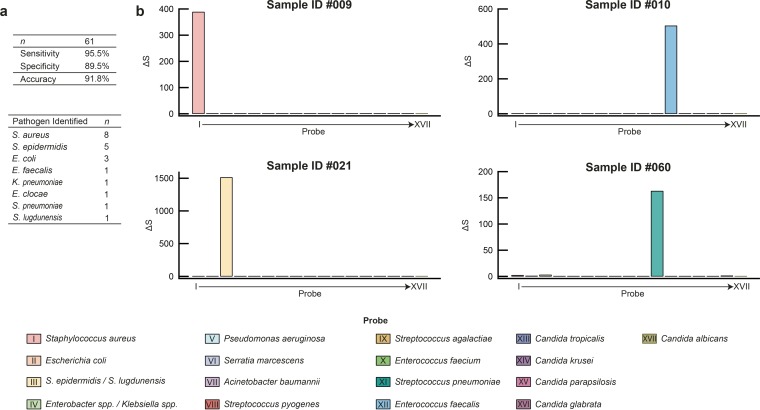
Clinical performance evaluation (a) Summary of clinical assay performance relative to blood culture and identity and frequency of pathogens detected by both the PID assay and culture. (b) Representative results for four culture-positive clinical specimens obtained with the PID assay.

We next evaluated the performance of our PID assay in comparison to conventional culturing using clinical specimens (see Materials and Methods). A total of 61 blood samples comprising culture-negative (*n* = 35) and culture-positive (*n* = 26) specimens, drawn into EDTA vacuettes, were collected consecutively over a period of 6 weeks. These samples were processed using procedures identical to the blood spike study described above. A comparison of results obtained via culturing with those via the PID assay, including discordant results, are shown in Table 1. Complete results of this study can be found in the Table S3 in the supplemental material. Summarized results and data for representative clinical samples are shown in Fig. 5 (remaining data can be found in Fig. S2 to S5 in the supplemental material). Notably, all clinical samples were assayed successfully, suggesting that, for the limited number of samples tested, neither common drug compounds administered to inpatients nor variation in leukocyte counts (ranging from 0.1 ×10^6^ to 25.2 ×10^6^ cells/ml, or roughly 1% to 350% of normal leukocyte counts) had a noticeable effect on PID assay performance. Of the 26 culture-positive samples, 22 samples contained pathogens covered by the PID assay panel, of which 21 cases were confirmed by the PID assay, correctly identifying the infecting pathogen in each case. Four specimens were culture positive for pathogens outside the PID panel and were indeed negative with the PID assay. Of the 35 specimens that were culture negative, 31 were also negative with the PID assay. Values for clinical sensitivity and specificity relative to blood culture were 95.5% and 89.5%, respectively, with an overall diagnostic accuracy of 91.8%. In 100% of cases where a culture was positive with a PID panel pathogen, the PID assay identified the same pathogen as was clinically identified.

**TABLE 1:** Comparison of results obtained via culturing and those from the PID assay

Sample ID	Blood culture result	PID assay result (peak signal Δ*S*)	Concordance	PCR/sequencing result^a^
001	*E. coli*	*E. coli* (197)	Yes	−
005	Coagulase-negative staphylococci	*S. epidermidis* group^b^ (37)	Yes	*S. epidermidis*
009	*S. aureus*	*S. aureus* (391)	Yes	−
010	*E. faecalis*	*E. faecalis* (506)	Yes	−
012	Negative	*S. epidermidis* group (174)	No	*S. hominis*
015	*S. aureus*	*S. aureus* (13,042)	Yes	−
017	Coagulase-negative staphylococci	*S. epidermidis* group (55)	Yes	*S. lugdunensis*
021	Coagulase-negative staphylococci	*S. epidermidis* group (1,518)	Yes	*S. epidermidis*
023	*E. coli*	*E. coli* (2,567)	Yes	−
024	*K. oxytoca*	*Klebsiella/Enterobacter* (145)	Yes	−
026	Coagulase-negative staphylococci	Negative	No	Negative
027	*E. clocae*	*Klebsiella/Enterobacter* (26)	Yes	−
030	Viridans group	Negative	Yes	−
031	Coagulase-negative staphylococci	*S. epidermidis* group (66)	Yes	*S. epidermidis*
032	Negative	*S. epidermidis* group (110)	No	*S. epidermidis*
035	*E. coli*	*E. coli* (5,396)	Yes	−
037	Negative	*S. aureus* (4,765)	No	*S. aureus*
039	*S. aureus*	*S. aureus* (110)	Yes	−
040	*S. aureus*	*S. aureus* (53)	Yes	−
043	Coagulase-negative staphylococci	*S. epidermidis* group (11,660)	Yes	*S. epidermidis*
045	Viridans group	Negative	Yes	−
046	*S. aureus*	*S. aureus* (12,008)	Yes	−
049	Negative	*S. aureus* (8,874)	No	*S. aureus*
051	*S. aureus*	*S. aureus* (9,455)	Yes	−
052	Unknown Gram negative	Negative	Yes	−
053	*Micrococcus* spp.	Negative	Yes	−
056	*S. aureus*	*S. aureus* (121)	Yes	−
059	Coagulase-negative staphylococci	*S. epidermidis* group (61)	Yes	*S. epidermidis*
060	*S. pneumoniae*	*S. pneumoniae* (164)	Yes	−
061	*S. aureus*	*S. aureus* (150)	Yes	−

a A negative γPNA assay result indicates that no panel pathogen was present but does not rule out the presence of other, less prevalent pathogens.

b The “*S. epidermidis* group” includes a large number of coagulase-negative staphylococci, such as but not limited to *S. epidermidis*, *S. hominis*, *S. lugdunensis*, and *S. haemolyticus*.

The five discordant cases were further examined. Sample 026 was deemed false negative by the PID assay, and samples 012, 032, 037, and 049 were deemed false positives by the PID assay. Sample 026 was positive by culture for *Staphylococcus epidermidis* but negative by the PID assay (Δ*S* = 0.03 for γPNA_CoNS_). *Staphylococcus*-specific 16S PCR confirmed that the sample processed by the PID assay was indeed negative. Subsequent review of patient records revealed that only a single-site blood draw was completed for two blood cultures. Of the two cultures, *S. epidermidis*, a common contaminant, was detected in one culture while the other remained negative. Since the culture was completed to clinically rule out a BSI, the culture results were deemed false positive, and no treatment was initiated. Of the four PID assay false positives, two (samples 012 and 032) were positive for *S. epidermidis*. The presence of *S. epidermidis* was indeed confirmed by *Staphylococcus*-specific 16S PCR and sequence analysis. Clinically, however, patient records did not corroborate our results, indicating that contamination was the likely cause. This interpretation is consistent with the fact that both samples 012 and 032 generated relatively low Δ*S* values for γPNA_CoNS_ of 174 and 110, respectively.

The remaining two false-positive PID assay results, generated with samples 037 and 049, were both positive for *S. aureus*. Interestingly, in contrast to *S. epidermidis*-positive samples 012 and 032, these *S. aureus*-positive samples generated very high peak Δ*S* values for γPNA_s. aureus_ of 4,765 and 8,874, respectively, corresponding to roughly 50 to 100 CFU/ml for *S. aureus*. Such high pathogen loads in samples 037 and 049 are unlikely to have been caused by contamination. Review of patient records revealed that sample 037 originated from a patient who 3 days prior had started treatment with elevated dosages of antibiotics based on *S. aureus*-positive blood cultures. Follow-up blood cultures still revealed an *S. aureus* infection, while subsequent culture results were negative (including those drawn in parallel to the sample tested with the PID assay). However, the results of the PID assay suggest that high numbers of intact *S. aureus* cells (free pathogen DNA is removed during early PID assay processing steps) were still circulating in the patient’s bloodstream, thus possibly indicating an ongoing infection and that the antibiotic inhibited growth, a well-known problem with blood cultures (32).

## DISCUSSION

Bloodstream infections are fast-acting with fatal consequences if treated improperly; hence, the time to appropriate antimicrobial intervention is the principal predictor of outcome (33, 34). Accurate and timely BSI diagnosis therefore plays a key role in disease control. Unfortunately, current diagnostic standards require unreasonably long turnaround times. This forces clinicians to resort to empirical treatment using broad-spectrum antimicrobial cocktails as a first-line response, a practice that leaves many patients improperly or inadequately treated and contributes to the rise of antimicrobial resistance (10). Molecular methods are better suited than culture-based methods for rapid diagnosis of BSIs, providing actionable information in time to guide initial treatment. We have presented an integrated process, capable of identifying a panel of 21 bacteria and fungi, directly from human blood samples in under 2.5 h, without the need to culture. The method is robust, fast, highly sensitive, and has been validated with clinical specimens. It thus has the potential to assist physicians in their decision-making process before culturing results become available.

Pathogen classification through rRNA encoding gene sequence recognition is well-established, and publicly available databases are common, comprehensive, and continuously updated. Despite the clinical need and the availability of information, a clear capability gap remains: a comprehensive diagnostic platform for pathogen identification without the need for culturing remains elusive. We demonstrate here the first practical diagnostic application of γPNAs in place of the ubiquitous DNA probes for superior identification and discrimination owing to their DNA-invading kinetics, high affinity, and exceptional sequence selectivity. We showed that even highly conserved sequences, such as the *Candida* 18S rDNA, which exhibits very limited interspecies variability and therefore renders DNA probes ill-suited, were successfully targeted using γPNAs. The largely negligible level of off-target binding exhibited by γPNAs enhanced detection sensitivity and increased confidence in result interpretation, thus enabling the development of a highly multiplexed assay covering a broad pathogen panel with substantially improved signal-to-background levels. The capabilities of γPNAs underscore their potential as an ideal tool for detection of nucleic acids where sensitive discrimination between large numbers of highly similar sequences is required. The utility of γPNAs as a diagnostic tool is further demonstrated in the combined detection of bacterial antibiotic resistance genes with species identification (see Fig. S6 in the supplemental material).

Our aim was to develop a fully integrated process for PID that could detect and discriminate a broad pathogen panel, thus maximizing its clinical utility. To this end, we leveraged γPNAs for their speed, sensitivity, and sequence selectivity on the analyte detection front. Likewise, we developed a modified sample preparation process that led to more robust results while simultaneously reducing the turnaround time to under 2.5 h, significantly faster than both blood culture (typically 1 to 3 days) and previously reported molecular assays (typically 5 to 12 h). Our PID platform successfully adopted several measures designed to increase sensitivity, reduce background, and facilitate the transition to a fluidic device. For instance, the effective depletion of hDNA from the sample allows the use of larger volumes of blood, directly increasing the total pathogen load assayed. Moreover, the process does not require centrifugation, simplifying future integration into a fluidic cassette. Sensitivity is further improved by a pathogen gDNA extraction/purification process that focuses on high-molecular-weight DNA, thereby reducing the level of copurified environmental contamination, which largely consists of low-molecular-weight DNA. Combined, these processes enable input of the entire sample into a single PCR, maximizing sensitivity. Downstream of these processes, our PCR process emphasizes the creation of long (~1.5-kbp) products, further lowering the likelihood of amplification from low-grade contaminating DNA. The resulting reduction in amplifiable background, combined with the resilience of γPNAs to off-target binding, proved highly capable, as demonstrated in the comprehensive blood spike study; all 21 panel pathogens were successfully detected at the single-CFU-per-milliliter level.

The PID platform was initially evaluated with either reference strains or clinical isolates of the 21 panel pathogens spiked into healthy donor blood at two load levels, <10 CFU/ml and <100 CFU/ml. All pathogens were detected consistently at both load levels; to the best of our knowledge, this has not been previously demonstrated with similarly broad pathogen panels. Performance assessment with clinical specimens highlighted the robustness of the system, based on the good positive and negative agreement with blood cultures. Moreover, the PID platform successfully detected the presence of an infection where antimicrobial intervention likely induced a negative blood culture. This highlights another advantage of molecular detection, since culture-based methods, inherently dependent on cell viability, are prone to false-negative results due to the potential prior use of antimicrobials. Moreover, the PID platform was specifically designed to only detect intact cells and not, for example, free pathogen DNA that may be circulating in a patient’s blood, as its clinical relevance remains an actively discussed topic (28).

We note that several other methods leveraging DNA probe-based detection (35, 36), sequencing (37), or electrospray-mass spectrometry (6) for pathogen identification directly from blood have been previously reported. However, these approaches have limitations with respect to panel size, sensitivity across the panel, level of detail, and time requirements, along with their propensity for false-positive results. The PID platform introduced here overcomes these issues. Most importantly, the PID platform convincingly demonstrates clinical utility, achieving impressive results when tested with clinical specimens, with equal to or better performance than conventional culturing methods in pathogen detection and identification. Further clinical evaluation of PID assay performance in comparison to culturing is required and will commence in a clinical setting with a fully automated fluidic device. We envision that upon successful automation, the integrated PID platform will be instrumental in significantly reducing the turnaround time needed to initiate a targeted treatment for BSIs, with far-reaching consequences in improving patient care.

## MATERIALS AND METHODS

### γPNA and PCR primer design.

For pathogen identification, target sequences were selected from either 16S (bacteria) or 18S (*Candida*) rRNA encoding sequences (NCBI nucleotide database). For each species, multiple sequences were aligned and consensus regions were identified, taking into account both intragenomic and intraspecies sequence variability for optimal sensitivity and coverage with a single γPNA oligomer. Within consensus regions, 15- to 17-mer γPNAs were designed to maximize mismatch energetics and minimize self-folding. Each γPNA was biotinylated, thereby enabling immobilization onto streptavidin-coated magnetic beads. For each target or target group, multiple γPNAs were designed in order to determine the optimal oligomer sequence, enabling uniform reaction conditions across the entire panel. Additional γPNAs were designed for the detection of carbapenem resistance-conferring genes, such as NDM-1 (New Delhi metallo-beta-lactamase) and KPC (*Klebsiella pneumoniae* carbapenemase) in Gram-negative bacteria. For this purpose, multiple *bla*_NDM-1_ and *bla*_KPC_ gene sequences from various bacterial species were retrieved from the NCBI database. Sequences were aligned, and conserved regions were selected for γPNA design following the rules outlined above (the γPNA sequences are reported in Table S1 in the supplemental material). For full-length 16S/18S PCR, amplification primers were based on published designs (38) or designed in-house by aligning the above-identified 16S and 18S sequences and identifying conserved regions for each. To avoid background amplification from human DNA, gene sequences for human 18S rDNA were included in the *Candida* sequence alignment and dissimilar regions were identified for primer design. All primers were equipped with a 5′-digoxin (Integrated DNA Technologies, Inc.). Primers used for *Staphylococcus*-specific 16S amplification were unlabeled. For amplification of resistance markers NDM-1 and KPC, primer sequences were derived from conserved regions identified in the multiple sequence alignments described above. The NDM-1-specific and KPC-specific primers were 5′-labeled with digoxin and used to generate amplicons of 669 bp and 687 bp, respectively. (Primer sequences can be found in Table S2 in the supplemental material.)

### Bacterial and fungal cultures.

Bacteria and fungi were obtained from the American Type Culture Collection (ATCC). Panel bacteria included *Acinetobacter baumannii* (ATCC 19606), *Enterobacter aerogenes* (ATCC 13048), *Enterobacter cloacae* (ATCC 13047), *Enterococcus faecalis* (ATCC 29212), *Enterococcus faecium* (ATCC 700221), *Escherichia coli* (ATCC BAA-2469), *Klebsiella oxytoca* (ATCC 49131), *Klebsiella pneumoniae* (ATCC BAA-1705 and ATCC BAA-2146), *Pseudomonas aeruginosa* (ATCC 110145), *Serratia marcescens* (ATCC 13880), *Staphylococcus aureus* (ATCC 43300), *Staphylococcus epidermidis* (ATCC 51625), *Staphylococcus lugdunensis* (ATCC 49576), *Streptococcus agalactiae* (ATCC 13813), *Streptococcus pneumoniae* (ATCC 6303), and *Streptococcus pyogenes* (ATCC 12344). Panel fungi consisted of *Candida albicans* (ATCC 90028), *Candida glabrata* (ATCC MYA-2950), *Candida krusei* (ATCC 14243), *Candida parapsilosis* (ATCC 90018), and *Candida tropicalis* (ATCC 13803). Bacteria were cultured in tryptic soy (TS) medium except for *S. pneumoniae*, for which TS was supplemented with 5% human blood. *Candida* spp. were cultured in Sabouraud dextrose medium. Liquid medium cultures for DNA extraction and blood spike experiments were seeded from single colonies, grown, and harvested at early to mid-log phase. For experiments requiring gDNA, microbial cells were harvested from cultures by centrifugation at 14,000 rpm for 1 min, and recovered cells were lysed by bead beating using the ZR Fungal/Bacterial DNA MicroPrep apparatus (Zymo Research) according to the manufacturer’s recommendations. Extracted gDNA was purified using a guanidine hydrochloride/silica spin column commercial kit. Final gDNA yield was quantified based on absorbance at 260 nm. For experiments which determined analytical sensitivity capabilities in blood, cultures were serially diluted in sterile phosphate-buffered saline (PBS). Diluted cultures (0.1-ml volume) were then spiked into 1.4 ml of freshly drawn human whole blood collected from healthy donors to generate a final volume of 1.5 ml and processed with the integrated PID assay (described below). For load assessment, diluted cell cultures were plated in triplicate and cultured as suggested by the ATCC until colonies could be quantified.

### PCR and γPNA bead assay.

For the PID assay, gDNA was amplified using the primers described in Table S2 in the supplemental material, enabling a pan-bacterial and/or a pan-*Candida* two-step amplification process. PCR was performed using Q5 Hot Start high-fidelity polymerase (New England Biolabs, Inc.) with the following cycling parameters: initial denaturation (98°C, 30 s); 4 cycles of denaturation (98°C, 10 s), annealing (62.5°C, 30 s), and extension (72°C, 45 s); 28 cycles of denaturation (94°C, 10 s) and annealing/extension (72°C, 45 s); a final incubation at 72°C for 2 min. The total number of cycles was experimentally optimized at 32, enabling single-cell detection with γPNAs for both bacteria and fungi, while maximizing the dynamic range. Roughly 6% of each PCR output was used directly for individual γPNA-mediated bead capture reactions. For γPNA-mediated bead capture, amplified DNA was incubated with biotin-labeled γPNA in 10 mM sodium phosphate buffer (pH 7.4) with 50 mM NaCl for 5 min at 85°C, thus enabling DNA to be selectively immobilized onto 1-µm MyOne C1 streptavidin-coated magnetic beads (Thermo Fisher Scientific, Inc.). Magnetic beads were then washed with 10 mM sodium phosphate buffer (pH 7.2) to remove nonspecifically bound products from the beads. Upon completion of the wash process, 175 fmol of an anti-digoxin–horseradish peroxidase conjugate (Jackson ImmunoResearch, Inc.) was added to each reaction mixture and the mixture was incubated for 5 min. Upon completion of the incubation, the beads were washed repeatedly. Chemiluminescence was induced with reagents provided in the SuperSignal enzyme-linked immunosorbent assay Femto Maximum Sensitivity kit (Thermo Fisher Scientific, Inc.) and quantified with a Promega GloMax 96 reader using white stripwell plates (Corning, Inc.). All measurements were performed in triplicate, and these data are expressed as means ± standard deviations (SD).

PCRs designed to elucidate the presence of amplified *Staphylococcus* 16S were performed under standard PCR conditions but using primers specific for *Staphylococcus* spp., where the diluted PCR product generated by the PID assay was used as input. PCR products were analyzed on agarose gels, and positive reaction products were sent for sequencing (Eton Bioscience, Inc.) and were analyzed using BLAST. Amplification of resistance markers NDM-1 and KPC was accomplished using a 3-plex primer mix consisting of resistance marker-specific primers and the universal bacterial 16S primers under standard PCR conditions. γPNAs specific for resistance markers and 16S genes from several Gram-negative pathogens were used for target detection following the protocol outlined above.

### Detection directly from blood.

Single-assay working volumes were 1.5 ml of whole blood. Complete details of HelixBind’s sample-processing steps are described elsewhere (WO/2016/044621). Briefly, human whole blood (EDTA treated), either pathogen spiked as described above or spiked with PBS, was mixed with a mild lysis solution comprised of a combination of detergents, which served to lyse human blood cells while not compromising the integrity of bacterial or fungal cells. After 1 min, a termination solution was added to prevent possible lysis of microbial cells. Magnetic beads, covalently modified with an anion exchanger, were added to selectively bind free DNA, consisting essentially of released human DNA. Beads were then immobilized and the mixture containing intact pathogens was transferred to a new vial. For microbial gDNA extraction, a lysis solution was added, based on a combination of multiple detergents and enzymatic components, where potentially contaminating DNA was rendered nonamplifiable prior to use (39, 40). Concurrently, magnetic beads, covalently modified with an anion exchanger, were added to selectively bind the released gDNA. After immobilization of the beads and removal of the supernatant, the beads were washed repeatedly in order to remove weakly bound impurities and short (<1-kbp) DNA fragments. Pathogen gDNA was eluted and concentrated using a size exclusion membrane (Millipore, Inc.). The gDNA was resuspended in 35 µl of water in preparation for the single-reaction PCR. This process ensures that the entire pathogen load present in 1.5 ml of human blood is input into a single PCR mixture, thereby maximizing sensitivity. The entire sample preparation process is completed in roughly 45 min. All spike-in measurements were performed in triplicate.

### Clinical samples.

This proof-of-concept study was approved by the Tufts Medical Center Institutional Review Board. Excess and discarded K_2_-EDTA-treated blood samples were collected from patients with suspected BSIs at the same time as specimens for blood culture were collected; samples for the PID assay analysis were stored at 4°C. Clinical samples were collected consecutively over a period of 6 weeks and selected for PID assay analysis if blood culture results were positive. No culture-positive samples were accepted in cases where less than 1.5 ml of the corresponding discarded blood volume was available. Time to positivity of culture-positive samples (*n* = 26) ranged from <13 to <145 h, with an average of approximately 37 h. Culture-negative blood samples (*n* = 35) were retrieved alongside culture-positive specimens and processed simultaneously with samples for the PID assay. Analysis of the clinical specimens was performed blinded with the integrated PID assay and subsequently compared to conventional blood culturing results. From each specimen, 1.5 ml of blood was processed for the PID assay and subjected to a single PCR followed by γPNA analysis with the entire probe panel.

### Data analysis.

All γPNA-based detection signals in this study are represented as the signal change (Δ*S*). In order to quantify the presence of a γPNA-mediated DNA amplicon, the following calculation was used: Δ*S* = (*S*^EXP^ − *S*^0^)/*S*^0^. *S*^EXP^ is defined as the experimentally determined optical signature (in relative light units [RLU]) in the presence of an analyte, and *S*^0^ is defined as the experimentally determined optical signature in the absence of an analyte. Optical signatures were measured on a Promega GloMax 96-well plate luminometer with an integration time of 2.5 s/well.

While the definition for *S*^EXP^ remained consistent throughout the study, *S*^0^ varied, depending on the nature of the experiment. For the experiments highlighted in Fig. 1b and c and 2, *S*^0^ was defined as the optical signature measured in the presence of blank sample (no DNA). For experiments highlighted in Fig. 1d and e, *S*^0^ was defined as the optical signature measured in the presence of a negative PCR control sample (no template added during PCR). For experiments highlighted in Fig. 3 and 4, *S*^0^ was defined as the typical optical signature measured in the presence of a sample which was derived from a whole-blood specimen spiked with PBS in a manner identical to the pathogen spike in experiments (*n* = 55). In the case of clinical specimens, *S*^0^ was defined as the typical optical signature measured from Tufts Medical Center excess culture-negative blood samples (*n* = 35). In order for a signal to be deemed positive, its Δ*S* value must be above 4× (SD) the background signal.

## SUPPLEMENTAL MATERIAL

Figure S1  Amplification of amplicons reduces effects of background contaminating DNA. Download Figure S1, PDF file, 0.1 MB

Figure S2  PID assay/culture-positive concordant results. Download Figure S2, PDF file, 0.4 MB

Figure S3 PID assay/culture-positive concordant results. Download Figure S3, PDF file, 0.4 MB

Figure S4 PID assay/culture-positive concordant results. Download Figure S4, PDF file, 0.2 MB

Figure S5  PID assay/culture-positive discordant results and culture-negative discordant results. Download Figure S5, PDF file, 0.4 MB

Figure S6  Tandem identification of bacterial species and antibiotic resistance-conferring genes. Download Figure S6, PDF file, 0.2 MB

Figure S7  Viability of *S. epidermidis* in EDTA blood at 4°C. Download Figure S7, PDF file, 0.1 MB

Table S1 Sequences of γPNA oligonucleotides used for bacterial, fungal, and resistance gene detection. Table S1, PDF file, 0.05 MB

Table S2 Sequences of PCR primers. Table S2, PDF file, 0.1 MB

Table S3 Performance assessment with clinical samples. Table S3, PDF file, 0.6 MB
