# Supplementary material for: Duplex DNA-Invading γ-Modified Peptide Nucleic Acids Enable Rapid Identification of Bloodstream Infections in Whole Blood
Source: mBio. 2016 Apr 19;7(2):e00345-16. doi: 10.1128/mBio.00345-16 (PMC4850259; doi:10.1128/mBio.00345-16)
Supplement: Figure S1 — Amplification of amplicons reduces effects of background contaminating DNA. Download [file mbo002162772sf1.pdf]

Amplification of kbp amplicons reduces effects of background contaminating DNA

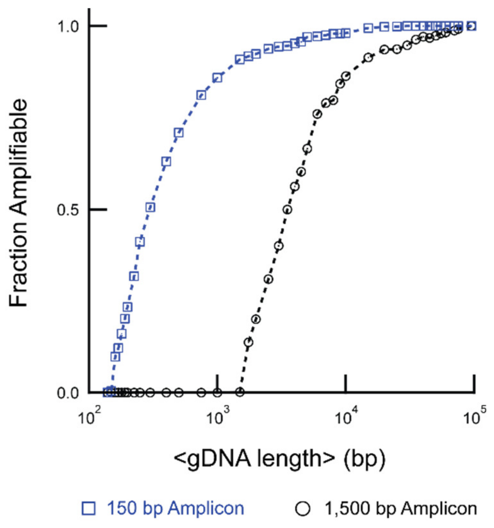

Figure S1 – Full-length 16S/18S rDNA genes of both bacteria and *Candida* serve as primary PCR target for the PID assay. This (long amplicon) approach has a number of advantages including the reduced likelihood of background amplification from environmental DNA as a result of using broad-range 16S rDNA PCR(*21, 41, 42*). Although the high specificity of γPNA probes alone ensures low background signal, the presence of random contaminating rDNA is expected to negatively impact overall PCR performance and potentially effect sensitivity. Since environmental DNA is usually of low quality (i.e. lower molecular weight) targeting larger amplicons is expected to significantly reduce the chance of background amplification. *In-silico* results depicted above (n = 10^4 simulations) demonstrate that, at a mean sheared size of 1.5 kbp, an amplification target of 150 bp, a size commonly used in rDNA diagnostic assays, is amplified in ~92.4% of the cases. In contrast, a 1.5 kbp amplification target, the size of the 16S rDNA amplicon in the PID assay, is amplified in only in 0.2% of the cases. Along similar lines, the use of duplex DNA cross-linking dyes which render DNA non-amplifiable (such as in(*39, 40*)) benefits from the use of long amplicons thereby further reducing background signal.
