# Supplementary material for: Duplex DNA-Invading γ-Modified Peptide Nucleic Acids Enable Rapid Identification of Bloodstream Infections in Whole Blood
Source: mBio. 2016 Apr 19;7(2):e00345-16. doi: 10.1128/mBio.00345-16 (PMC4850259; doi:10.1128/mBio.00345-16)
Supplement: Figure S2 — PID assay/culture-positive concordant results. Download [file mbo002162772sf2.pdf]

PID assay / Culture positive concordant results

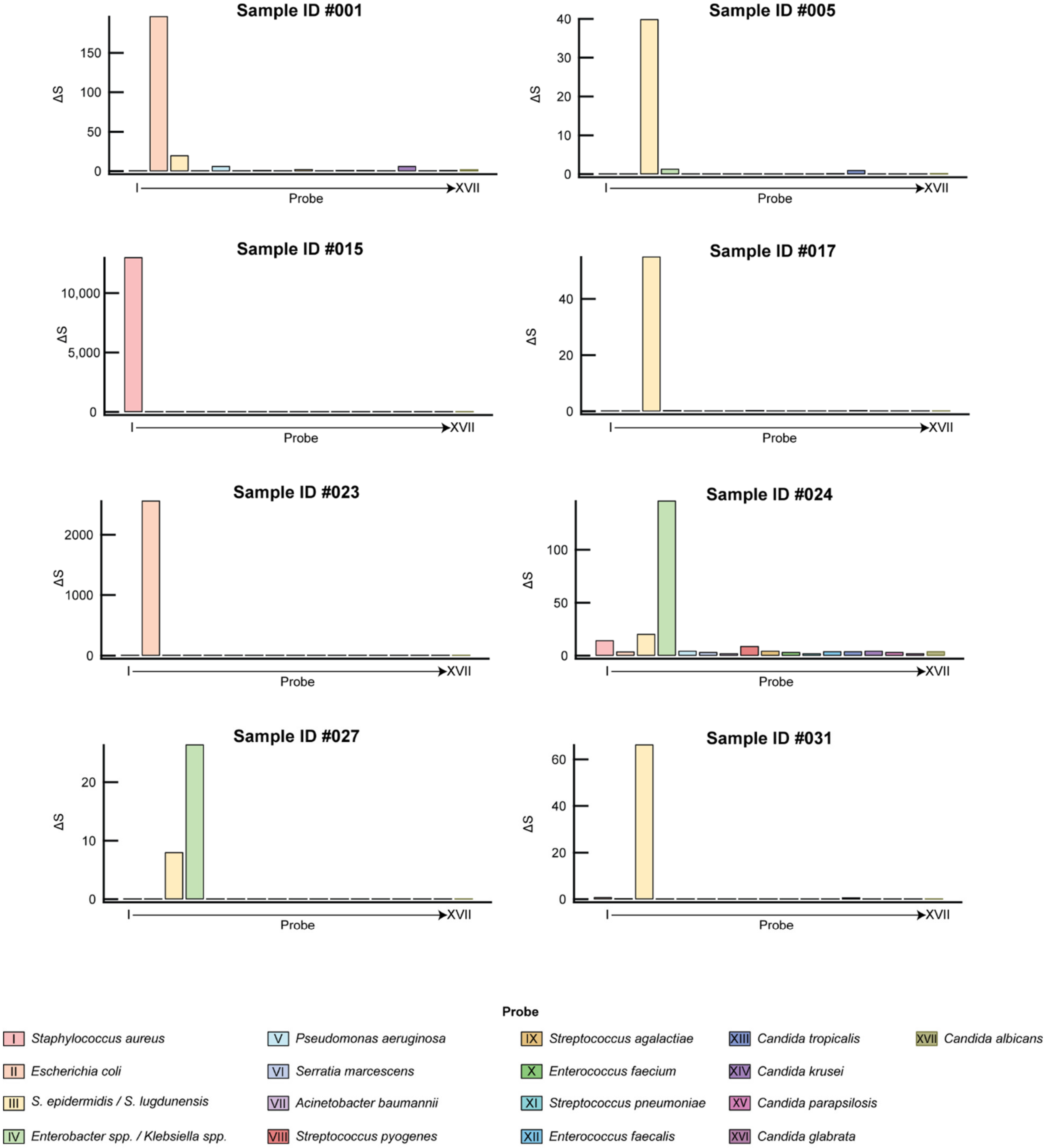

Figure S2 - Performance of PID assay with concordant culture positive clinical specimens. Sample numbers refer to patient specimens listed in Tables 1 and S3. Sample ID #001, #005, #015, #017, #023, #024, #027, #031 were deemed positive for *E. coli*, CoNS, *S. aureus*, CoNS, *E. coli*, *Klebsiella spp. /Enterobacter spp*,, *Klebsiella spp. /Enterobacter spp*, and CoNS, respectively.
