# Supplementary material for: Duplex DNA-Invading γ-Modified Peptide Nucleic Acids Enable Rapid Identification of Bloodstream Infections in Whole Blood
Source: mBio. 2016 Apr 19;7(2):e00345-16. doi: 10.1128/mBio.00345-16 (PMC4850259; doi:10.1128/mBio.00345-16)
Supplement: Figure S3 — PID assay/culture-positive concordant results. Download [file mbo002162772sf3.pdf]

**PID assay / Culture positive concordant results**

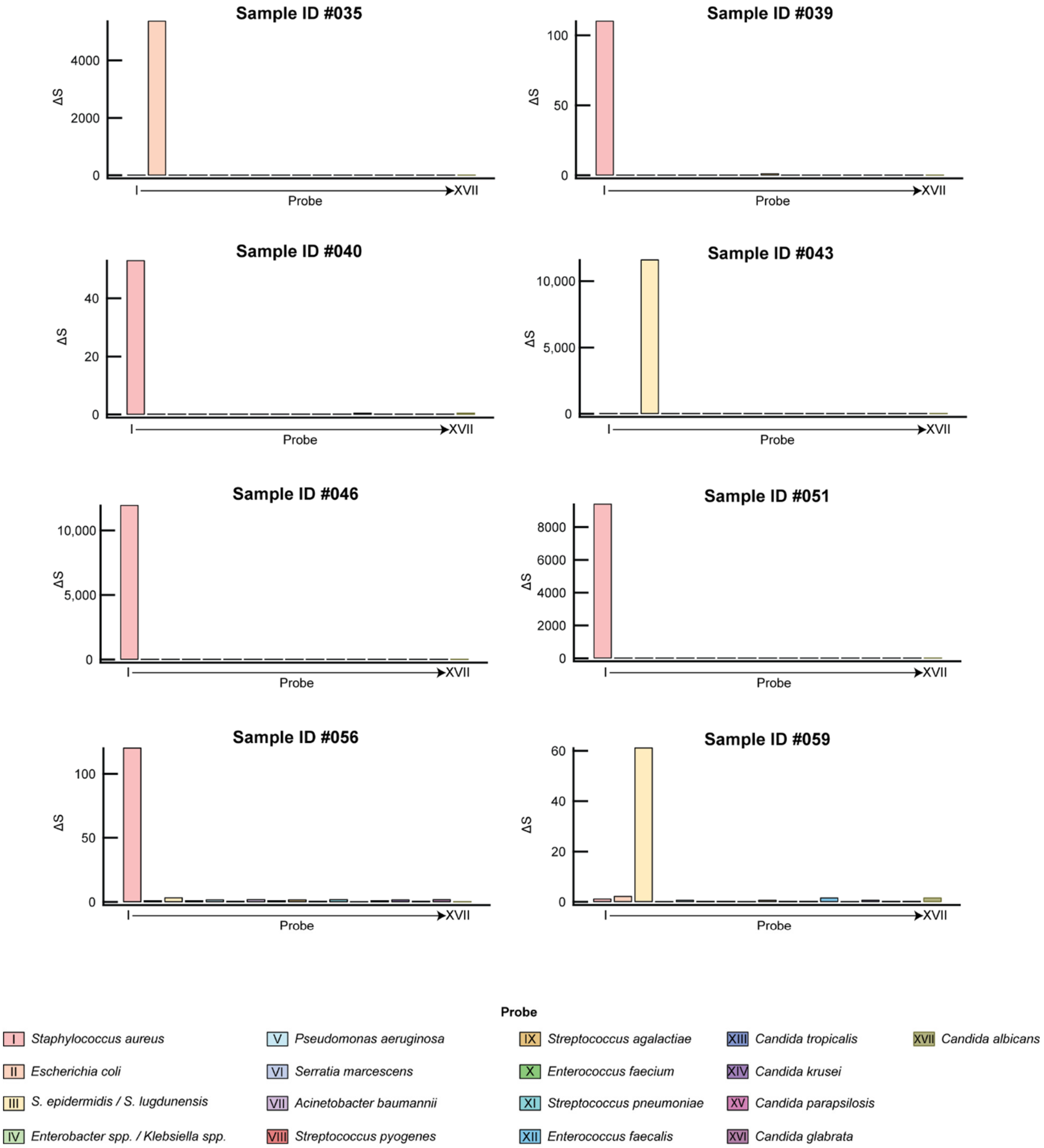


**Figure S3** - Performance of PID assay with concordant culture positive clinical specimens. Sample numbers refer to patient specimens listed in Tables 1 and S3. Sample ID #035, #039, #040, #043, #046, #051, #056, #059 were deemed positive for *E. coli*, *S. aureus*, *S. aureus*, CoNS, *S. aureus*, *S. aureus*, *S. aureus*, and CoNS, respectively.
