# Supplementary material for: Duplex DNA-Invading γ-Modified Peptide Nucleic Acids Enable Rapid Identification of Bloodstream Infections in Whole Blood
Source: mBio. 2016 Apr 19;7(2):e00345-16. doi: 10.1128/mBio.00345-16 (PMC4850259; doi:10.1128/mBio.00345-16)
Supplement: Figure S4 — PID assay/culture-positive concordant results. Download [file mbo002162772sf4.pdf]

PID assay / Culture positive concordant results

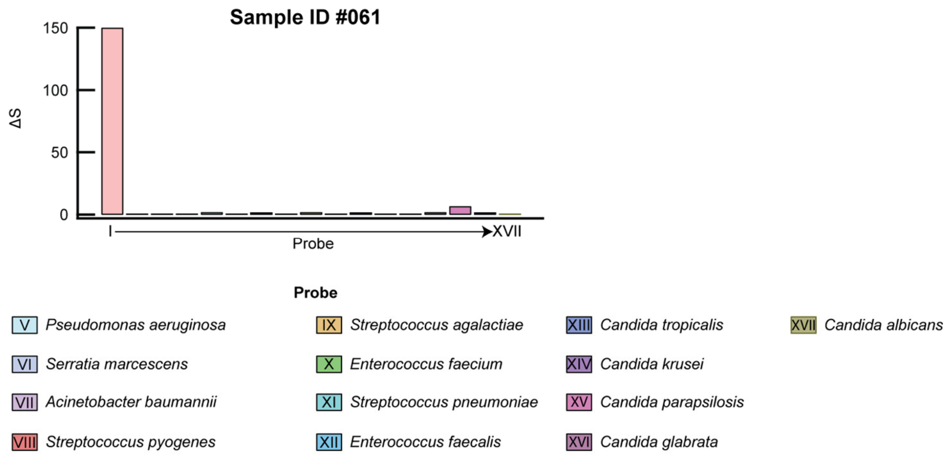

Figure S4 - Performance of PID assay with concordant culture positive clinical specimens. Sample number refers to patient specimen listed in Tables 1 and S3. Sample ID #061 was deemed positive for *S. aureus*.
