# Supplementary material for: Duplex DNA-Invading γ-Modified Peptide Nucleic Acids Enable Rapid Identification of Bloodstream Infections in Whole Blood
Source: mBio. 2016 Apr 19;7(2):e00345-16. doi: 10.1128/mBio.00345-16 (PMC4850259; doi:10.1128/mBio.00345-16)
Supplement: Figure S5 — PID assay/culture-positive discordant results and culture-negative discordant results. Download [file mbo002162772sf5.pdf]

PID assay / Culture positive discordant results

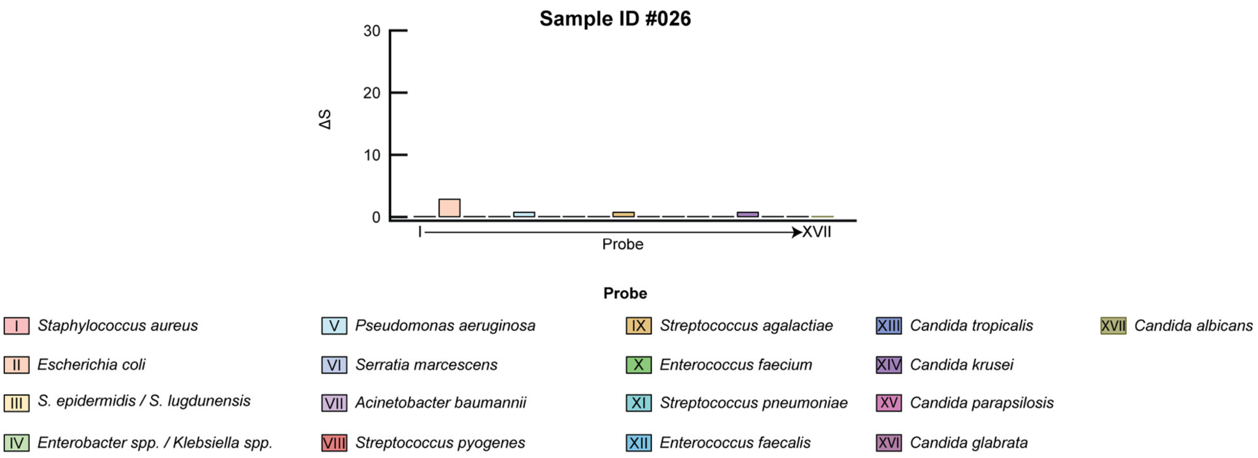

Figure S5a - Performance of PID assay with discordant (false negative) clinical specimens. Sample number refers to patient specimen listed in Tables 1 and S3.

PID assay / Culture negative discordant results

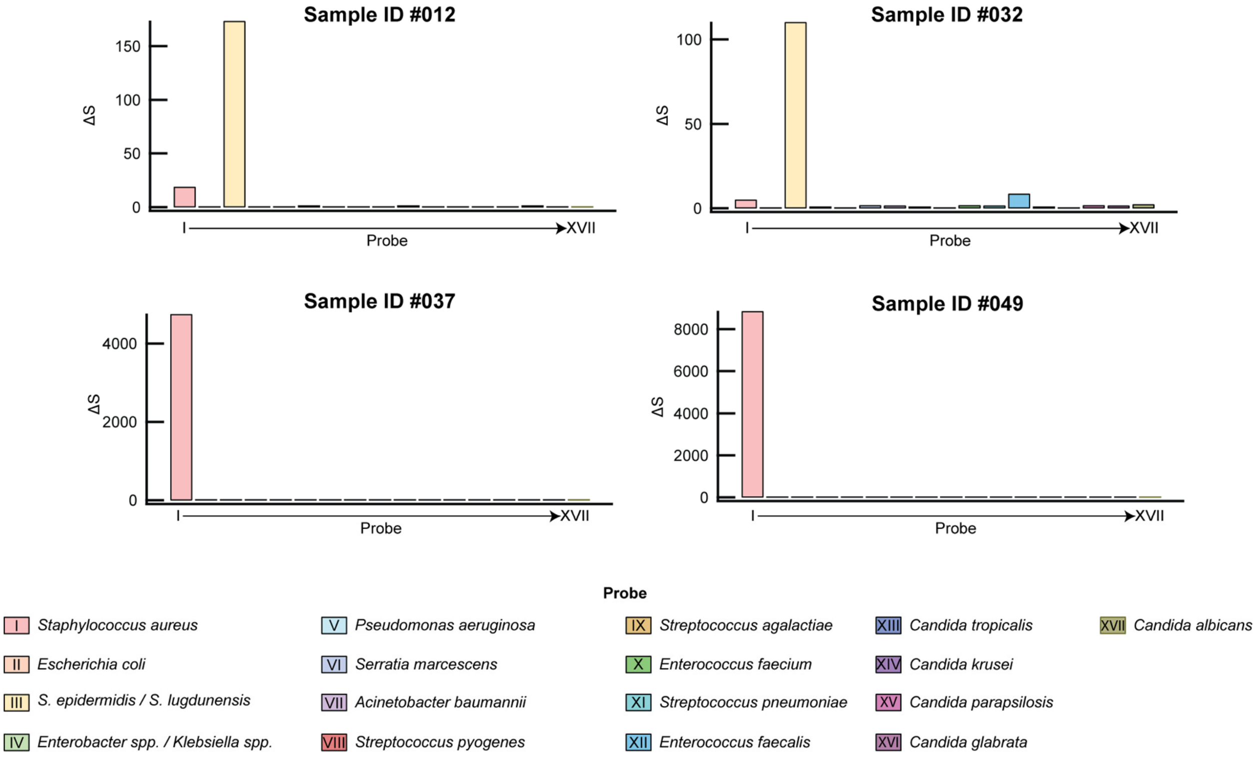

Figure S5b - Performance of PID assay with discordant (false positive) clinical specimens. Sample numbers refer to patient specimens listed in Tables 1 and S3.
