# Supplementary material for: Duplex DNA-Invading γ-Modified Peptide Nucleic Acids Enable Rapid Identification of Bloodstream Infections in Whole Blood
Source: mBio. 2016 Apr 19;7(2):e00345-16. doi: 10.1128/mBio.00345-16 (PMC4850259; doi:10.1128/mBio.00345-16)
Supplement: Figure S6 — Tandem identification of bacterial species and antibiotic resistance-conferring genes. Download [file mbo002162772sf6.pdf]

Tandem identification of bacterial species and antibiotic resistance conferring genes

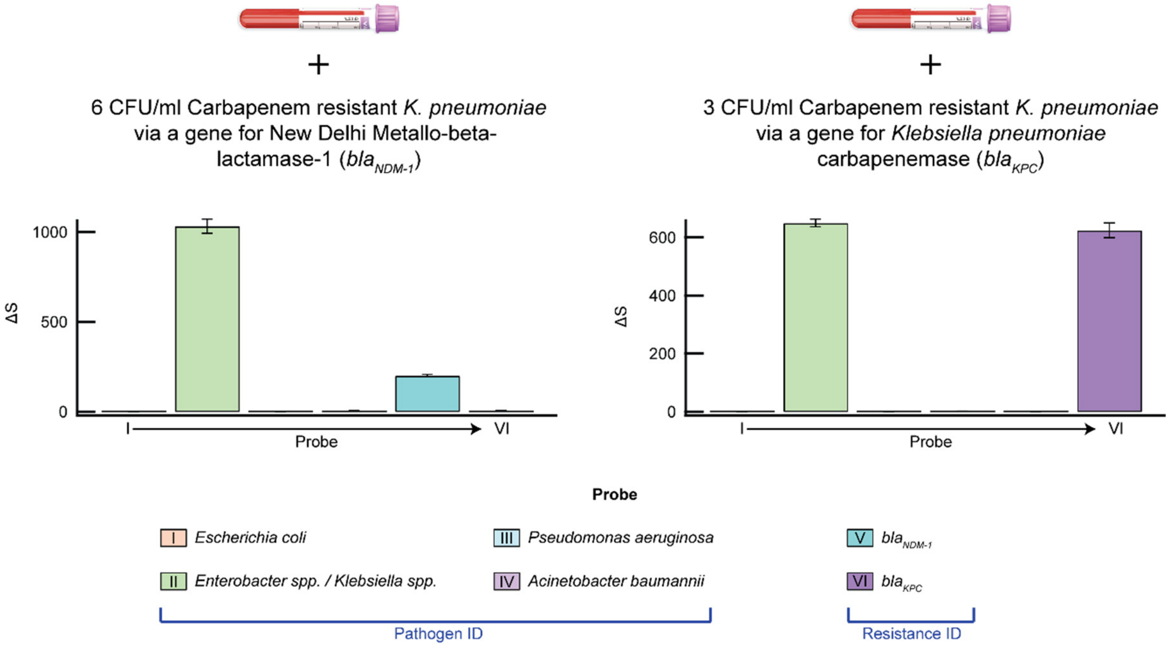

Figure S6 – Detection of carbapenem resistance genes and simultaneous pathogen identification from blood. Blood (1.5 ml) was spiked with the indicated CFU/ml levels of *K. pneumoniae* strains BAA-2146 (NDM-1 resistance marker) and BAA-1705 (KPC resistance marker), respectively, and the samples were processed according to the standard protocol used for the PID assay. Following extraction, the gDNA was subjected to multiplex-PCR using primers specific for $bla_{\text{NDM-1}}$, bla_{KPC} and 16S rDNA. Amplified DNA was assayed with γPNA probes specific for 16S rDNA from four Gram negative bacteria (probes I – IV) and resistance markers (probes V and VI) using the standard protocol as outlined in the main text. Experiments were completed in triplicate where data is represented as mean ± s.d.
