# Supplementary material for: Duplex DNA-Invading γ-Modified Peptide Nucleic Acids Enable Rapid Identification of Bloodstream Infections in Whole Blood
Source: mBio. 2016 Apr 19;7(2):e00345-16. doi: 10.1128/mBio.00345-16 (PMC4850259; doi:10.1128/mBio.00345-16)
Supplement: Figure S7 — Viability of S. epidermidis in EDTA blood at 4°C. Download [file mbo002162772sf7.pdf]

Viability of *S. epidermidis* in EDTA blood at 4°C

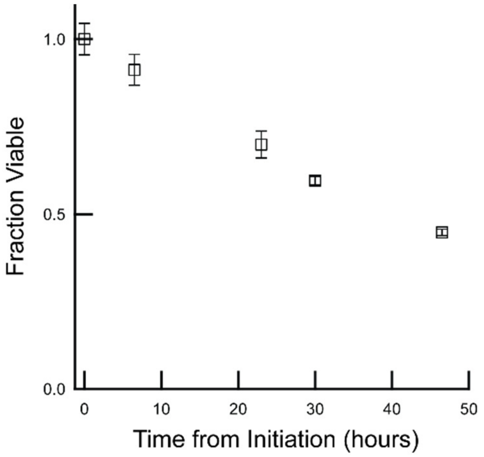

Figure S7 –Clinical sample processing with the PID assay was performed with excess and discarded patient samples that were stored for up to 48h at 4ºC in EDTA vacuettes. To assess the effect of this process on cell viability, a mid-log phase diluted culture of a common pathogen, *S. epidermidis*, was spiked into a freshly drawn human blood sample in an EDTA vacuette and incubated at 4°C for up to 48h. At indicated time intervals 100 µl was plated and the resulting colony count compared to that generated at the time of spike. The results shown above indicate a gradual loss in viability of about 50% over 48 hrs. Extrapolating these results to storage conditions of clinical specimens tested in this study suggests that the impact of a delay in sample processing by up to 48 hours likely has a marginal impact on pathogen detection. Plating experiments were completed in triplicate where data is presented as mean ± s.d.
