# Supplementary material for: Duplex DNA-Invading γ-Modified Peptide Nucleic Acids Enable Rapid Identification of Bloodstream Infections in Whole Blood
Source: mBio. 2016 Apr 19;7(2):e00345-16. doi: 10.1128/mBio.00345-16 (PMC4850259; doi:10.1128/mBio.00345-16)
Supplement: Table S1 — Sequences of γPNA oligonucleotides used for bacterial, fungal, and resistance gene detection. [file mbo002162772st1.pdf]

Supplemental Table S1: Sequences of γPNA oligos used for bacterial, fungal, and resistance detection

Group	Target Organism or Gene	Sequence (C to N terminus)
Gram Positive	*Staphylococcus aureus*	TCGAAGAGCAGGCAA
	Staphylococcus epidermidis<br>*Staphylococcus lugdunensis*	TCGAGGTTTACCAATG
	Enterococcus faecalis	AAGTCAATGATTGCAGG
	Enterococcus faecium	TTGTCAATGAGAGTAGG
	Streptococcus agalactiae	TACACAATTAATGAGAA
	Streptococcus pyogenes	GCAATCAGAGAGAATA
	Streptococcus pneumoniae	TCGGATGATACCAATT
Gram Negative	*Escherichia coli*	CAGTTACTCGTTTCCAT
	Pseudomonas aeruginosa	CGCGGTGATTCTAGAGT
	Serratia marcescens	AATTCAAGTGGTGGAA
	Acinetobacter baumannii	GGTGATAGAGATCCAT
	Enterobacter aerogenes<br>*Enterobacter cloacae*<br>*Klebsiella oxytoca*<br>*Klebsiella pneumoniae*	CTCGTTCGAGAGACAC
Fungal	*Candida albicans*	GTATTTACCGATGGG
	Candida glabrata	ACGTAAGGTCATGTGC
	Candida krusei	GATCTAAAAGGTGCC
	Candida tropicalis	TCAGGCTTCTGTAAC
	Candida parapsilosis	TGCGTAGTTTTTCTA
Resistance Gene	*bla*$_{NDM-1}$	ACCAAGCTGTTGCGTAAC
	bla$_{KPC}$	AGTACGGACAACAGTCT
