# Supplementary material for: Duplex DNA-Invading γ-Modified Peptide Nucleic Acids Enable Rapid Identification of Bloodstream Infections in Whole Blood
Source: mBio. 2016 Apr 19;7(2):e00345-16. doi: 10.1128/mBio.00345-16 (PMC4850259; doi:10.1128/mBio.00345-16)
Supplement: Table S2 — Sequences of PCR primers. [file mbo002162772st2.pdf]

Supplemental Table S2: Sequences of PCR Primers

Target	Forward Target Sequence (5' to 3')	Reverse Target Sequence (5' to 3')
Bacterial 16S rDNA	AGAGTTTGATC**X**TGGCTCAG	GG**Y**TACCTTGTTACGACTT
Fungal 18S rDNA	TGGCTCATTAAATCAGTTATCGT	GTCTGGACCTGGTGAGTTTC
Staphylococcus 16S rDNA	GATAACTTCGGGAAACCGGA	GG**Y**TACCTTGTTACGACTT
$bla_{\text{NDM-1}}$	GATTGGCCAGCAAATGGAA	GGCTCATCACGATCATGCT
bla_{KPC}	TCGCTAAACTCGAACAGGA	CGCTGTGCTTGTCATCCT

*Bases in bold lettering: X = A/C, Y = C/T. Each primer has a 6-7 mer random C/T tail added to the 5' end to enable a more rapid two-step PCR process.
