# Supplementary material for: Duplex DNA-Invading γ-Modified Peptide Nucleic Acids Enable Rapid Identification of Bloodstream Infections in Whole Blood
Source: mBio. 2016 Apr 19;7(2):e00345-16. doi: 10.1128/mBio.00345-16 (PMC4850259; doi:10.1128/mBio.00345-16)
Supplement: Table S3 — Performance assessment with clinical samples. [file mbo002162772st3.pdf]

Supplemental Table S3: Performance assessment with clinical samples

Sample ID	Blood Culture	γPNA assay (peak signal, ΔS)	Concordance	PCR/Sequencing
001	*E. coli*	*E. coli* (197)	Yes	-
002	Negative	Negative	Yes	-
003	Negative	Negative	Yes	-
004	Negative	Negative	Yes	-
005	Coagulase Negative Staphylococci	*S. epidermidis* group (37)	Yes	*S. epidermidis*
006	Negative	Negative	Yes	-
007	Negative	Negative	Yes	-
008	Negative	Negative	Yes	-
009	*S. aureus*	*S. aureus* (391)	Yes	-
010	*E. faecalis*	*E. faecalis* (506)	Yes	-
011	Negative	Negative	Yes	-
012	Negative	*S. epidermidis* group (174)	No	*S. hominis*
013	Negative	Negative	Yes	-
014	Negative	Negative	Yes	-
015	*S. aureus*	*S. aureus* (13,042)	Yes	-
016	Negative	Negative	Yes	-
017	Coagulase Negative Staphylococci	*S. epidermidis* group (55)	Yes	*S. lugdunensis*
018	Negative	Negative	Yes	-
019	Negative	Negative	Yes	-
020	Negative	Negative	Yes	-
021	Coagulase Negative Staphylococci	*S. epidermidis* group (1,518)	Yes	*S. epidermidis*
022	Negative	Negative	Yes	-
023	*E. coli*	*E. coli* (2,567)	Yes	-
024	*K. oxytoca*	*Klebsiella/Enterobacter* (145)	Yes	-
025	Negative	Negative	Yes	-
026	Coagulase Negative Staphylococci	Negative	No	Negative
027	*E. clocae*	*Klebsiella/Enterobacter* (26)	Yes	-
028	Negative	Negative	Yes	-
029	Negative	Negative	Yes	-
030	Viridans group	Negative	Yes	-
031	Coagulase Negative Staphylococci	*S. epidermidis* group (66)	Yes	*S. epidermidis*
032	Negative	*S. epidermidis* group (110)	No	*S. epidermidis*
033	Negative	Negative	Yes	-
034	Negative	Negative	Yes	-
035	*E. coli*	*E. coli* (5,396)	Yes	-
036	Negative	Negative	Yes	-
037	Negative	*S. aureus* (4,765)	No	*S. aureus*
038	Negative	Negative	Yes	-
039	*S. aureus*	*S. aureus* (110)	Yes	-
040	*S. aureus*	*S. aureus* (53)	Yes	-
041	Negative	Negative	Yes	-
042	Negative	Negative	Yes	-
043	Coagulase Negative Staphylococci	*S. epidermidis* group (11,660)	Yes	*S. epidermidis*
044	Negative	Negative	Yes	-
045	Viridans group	Negative	Yes	-
046	*S. aureus*	*S. aureus* (12,008)	Yes	-
047	Negative	Negative	Yes	-
048	Negative	Negative	Yes	-
049	Negative	*S. aureus* (8,874)	No	*S. aureus*
050	Negative	Negative	Yes	-
051	*S. aureus*	*S. aureus* (9,455)	Yes	-
052	Unknown Gram Negative	Negative	Yes	-
053	*Micrococcus*	Negative	Yes	-
054	Negative	Negative	Yes	-
055	Negative	Negative	Yes	-
056	*S. aureus*	*S. aureus* (121)	Yes	-
057	Negative	Negative	Yes	-
058	Negative	Negative	Yes	-
059	Coagulase Negative Staphylococci	*S. epidermidis* group (61)	Yes	*S. epidermidis*
060	*S. pneumoniae*	*S. pneumoniae* (164)	Yes	-
061	*S. aureus*	*S. aureus* (150)	Yes	-

Note: A 'negative' γPNA assay indicates that no panel pathogen is present but does not rule out the presence of other, less prevalent pathogens.
'*S. epidermidis* group' includes a large number of Coagulase Negative Staphylococci such as, but is not limited to, *S. epidermidis*, *S. hominis*, *S. lugdunensis*, and *S. haemolyticus*.
